# Estimation of physiologic ability and surgical stress (E-PASS) predicts postoperative complications after adrenalectomy

**DOI:** 10.1007/s13304-025-02145-w

**Published:** 2025-03-03

**Authors:** Mehmet Vehbi Kayra, Mehmet Eflatun Deniz, Cevahir Ozer, Sibel Catalca, Serdar Toksoz, Hakan Yabanoglu

**Affiliations:** 1 Faculty of Medicine, Department of Urology, Baskent University, Dadaloglu Mh Serinevler 2591 Sk No: 4/A Yuregir, 01250 Adana, Turkey; 2 Faculty of Medicine, Department of Anesthesiology, Baskent University, Adana, Turkey; 3 Department of Urology, Sincan Training and Research Hospital, Gokcek, 250Th Street No: 2/A Sincan, 06949 Ankara, Turkey; 4 Faculty of Medicine, Department of General Surgery, Baskent University, Adana, Turkey

**Keywords:** Adrenalectomy, E-PASS, Adrenal mass, Complications

## Abstract

The Estimation of Physiologic Ability and Surgical Stress (E-PASS) score, initially developed for gastrointestinal surgery, is a validated system used to predict postoperative complications by evaluating preoperative and intraoperative factors. This study aims to assess the effectiveness of the E-PASS score in predicting postoperative complications following adrenalectomy. In this single-center retrospective study, we analyzed data from 202 patients who underwent adrenalectomy by a single surgeon between January 2017 and March 2024. 182 patients with complete data and who met the study criteria were included in the study. Demographic, clinical, intraoperative, and postoperative data were collected and analyzed, including preoperative complaints, ASA classification, ECOG performance status, presence of systemic diseases, type of surgery, and intraoperative details, such as blood loss and complications. Postoperative complications were classified using the Clavien–Dindo Classification. The mean age of the patients was 48.7 ± 13.6 years. The mean BMI was 24.1 kg/m^2^. Postoperative complications were observed in 26.4% of patients, categorized as Grade 1 (54.1%), Grade 2 (25%), Grade 3 (16.7%), and Grade 4 (4.2%). Multivariate logistic regression identified higher BMI (OR = 1.394) and an E-PASS CRS score > − 0.0677 (OR = 6.17) as independent risk factors for complications. ROC curve analysis determined this CRS score cut-off with an AUC of 0.866 (CI 0.808–0.923; *p* < 0.001). The E-PASS scoring system effectively predicts postoperative complications in adrenalectomy. Its integration into clinical practice can enhance the identification of high-risk patients, optimize perioperative management, and potentially reduce adverse outcomes.

## Introduction

Adrenalectomy is indicated for a variety of conditions including adrenal cancers, Conn’s syndrome (CS), Cushing's syndrome (CuS), pheochromocytomas (PHEO), adrenal metastasis and asymptomatic adenomas larger than a certain size [1]. Depending on the size and nature of the adrenal lesion, as well as the patient's overall health status, the surgery can be performed using either an open or laparoscopic approach [2]. Both open adrenalectomy (OA) and laparoscopic adrenalectomy (LA) are complex procedures that while generally safe, carry a risk of complications [3, 4]. The risk is heightened due to the proximity of the adrenal glands to major blood vessels and the potential for significant hormonal fluctuations during and after the procedure, which can lead to hemodynamic instability and other perioperative challenges [5–7]. These complications can arise from the surgical technique itself or from the patient's physiological response to the stress of surgery, necessitating careful preoperative assessment and postoperative monitoring [3, 7].

Various risk calculators have been successfully used to predict the risk of complications after adrenalectomy and several nomograms have also been developed [8–10]. However, there is no scoring system in the literature that comprehensively addresses both preoperative physical condition and intraoperative risk variables. The Estimation of Physiologic Ability and Surgical Stress (E-PASS) score, originally developed for use in gastrointestinal (GI) surgery, is a validated scoring system that thoroughly evaluates both preoperative condition of the patient and intraoperative factors to predict the risk of postoperative complications [11]. However, studies have shown that the E-PASS score also provides a high level of accuracy in predicting postoperative complications in surgeries beyond the GI surgery [12–14]. Therefore, we aimed to evaluate the effectiveness of the E-PASS scoring system in predicting postoperative complications following adrenalectomy.

## Methods

This study was approved by the Baskent University Medical and Health Sciences Research Committee (Project No: KA24/69) and conducted in accordance with the principles of the Declaration of Helsinki.

### Methodology and criteria for selecting patients

In this single-center study, 202 cases of OA and LA performed by a single surgeon with experience in endocrine surgery between January 2017 and March 2024 were retrospectively reviewed from the hospital information system. Patients who underwent additional surgeries simultaneously with adrenalectomy were excluded from the study. Due to incomplete data or failure to meet the study criteria, 20 patients were excluded and a total of 182 patients were included in the study.

The demographic characteristics of the patients, including age, gender and body mass index (BMI) were recorded. Preoperative, intraoperative and postoperative findings of all patients in our study were examined. Patient complaints at presentation, clinical diagnosis, lesion side and size, history of abdominal surgery, physical status classification by the American Society of Anesthesiologists (ASA), performance score by the Eastern Cooperative Oncology Group (ECOG), the presence of additional systemic diseases and type of surgery were evaluated. Intraoperative findings, such as procedure duration, amount of blood loss, need for blood transfusion and intraoperative complications were recorded. Postoperative hospital stay, drain use and postoperative complications were identified. Postoperative complications were categorized according to the Clavien–Dindo Classification (CDC). [15] The E-PASS score was calculated using the current findings [11].

### E-PASS score

The E-PASS score is a surgical risk evaluation method that includes the Preoperative Risk Score (PRS), the Surgical Stress Score (SSS) and the Comprehensive Risk Score (CRS) derived from them. This scoring system was created by Haga and colleagues to assess the risk of postoperative complications in elective GI surgeries [11]. The formulas and parameters used to calculate PRS, SSS and CRS are shown in Table 1.

**Table 1:** Calculation of preoperative risk, surgical stress and comprehensive risk scores

Score	Formula
PRS	−0.0686 + 0.00345X1 + 0.323X2 + 0.205X3 + 0.153X4 + 0.148X5 + 0.0666X6
SSS	−0.342 + 0.0139Y1 + 0.0392Y2 + 0.352Y3
CRS	−0.328 + 0.936(PRS) + 0.976(SSS)

Age, serious heart disease, serious pulmonary disease, and diabetes mellitus are defined as present 1 or absent 0

*PRS* Preoperative Risk Score, *X1* age, *X2* serious heart disease, *X3* serious pulmonary disease, *X4* diabetes mellitus, *X5* performance status (0–4), *X6* ASA score (1–5), *SSS* Surgical Stress Score, *Y1* blood loss/body weight (g/kg), *Y2* operating time (hour), *Y3* skin incision length (0: minor incision, 1: laparotomy or thoracotomy only, 2: laparotomy and thoracotomy) *CRS* Comprehensive Risk Score

### Premedication

In patients undergoing adrenalectomy, preoperative management was tailored according to the clinical diagnosis, following evaluation in a multidisciplinary council that included endocrinologists. For those with PHEO, alpha-adrenergic blockade was administered to control blood pressure and prevent intraoperative hypertensive crises. In patients with CuS, glucocorticoid therapy was applied to manage adrenal insufficiency risks anticipated during the intraoperative period. For CS, potassium supplementation and aldosterone antagonists were used to correct hypokalemia and control hypertension prior to surgery.

### Surgical technique for open adrenalectomy

The patient was positioned in a lateral decubitus position. A flank or midline incision was made to provide access to the adrenal gland. Dissection was performed to expose the adrenal gland, with careful separation of surrounding tissues. The main adrenal vessels, including the adrenal vein and arteries were identified and ligated to control bleeding. The adrenal gland was then dissected free and removed. A drain was placed in all cases.

### Surgical technique for laparoscopic adrenalectomy

The patient was positioned in a lateral decubitus position. Transperitoneal surgery was performed. Pneumoperitoneum was established using a Veress needle for carbon dioxide insufflation. Generally, three trocars were used. Dissection was performed to free the adrenal gland from surrounding tissues. The main adrenal vessels were identified secured using clips and transected. The adrenal gland was then removed through one of the trocar sites using a retrieval bag. A drain was placed if deemed necessary.

### Statistical analysis

Data coding and statistical analyses were performed on the computer using the SPSS 22 software package program (IBM SPSS Statistics, IBM Corporation, Chicago, IL). The normality of the variables was assessed using the Shapiro–Wilk test. Normally distributed variables are presented as mean ± standard deviation, while non-normally distributed variables are reported as median (interquartile range). An independent sample T test, or Mann–Whitney U test, was used for non-categorical variables. For categorical variables, Chi-square or Fisher's exact tests were used. The predictive ability of CRS for postoperative complications in patients undergoing RC was assessed using the receiver operating characteristic (ROC) curve with a 95% confidence interval. Postoperative complications were evaluated using the backward LR method and multivariate logistic regression analysis for patients who were not included in the CRS calculation of variables that showed statistically significant differences between developing and non-developing patients. Multivariate analysis identified independent risk factors for postoperative complications. A p value of less than 0.05 was considered statistically significant.

## Results

The mean age of the 182 patients who underwent adrenalectomy was 48.7 ± 13.6 years. Of these patients, 116 (63.7%) were female. The mean body mass index (BMI) of the patients was 24.1 kg/m^2^. The most common presenting complaint was nonspecific pain (58.3%) and the most frequent clinical diagnosis was incidentaloma (26.4%). The median lesion diameter was 42 mm (IQR: 27.7–60), and 95 masses (52.2%) were functional (Table 2). Laparoscopic surgery was performed in 101 patients (55.5%). Conversion from laparoscopic to open surgery occurred in 37 patients (20.3% of total and 36.6% of laparoscopic cases), with intraoperative bleeding being the most common cause. Postoperative complications were observed in 48 patients (26.4%) (Table 3). According to the Clavien–Dindo classification system, complications were categorized as follows: Grade 1 in 26 patients (54.1%), Grade 2 in 12 patients (25%), Grade 3 in 8 patients (16.7%), and Grade 4 in 2 patients (4.2%). Detailed data on postoperative complications are presented in Table 4.

**Table 2:** Comparative analysis of demographic, clinical, radiological and laboratory data

	Total (*n* = 182)	Presented with no postoperative complications (*n* = 134, % 73.6)	Presented with postoperative complications (*n* = 48, % 26.4)	*p*
Demographic Data				
Age (Mean ± SD)	48.7 ± 13.6	48 ± 13.6	50 ± 13.6	0.929^t^
Female Gender, *n* (%)	116 (63.7)	90 (67.2)	26 (54.2)	0.108^c^
BMI (kg/m^2^) (Median)(IQR)	24.1 (23–25.7)	23.7 (22.9–24.9)	25.6 (23.7–27.8)	** < 0.001**^m^
Clinical Data				
Presenting Symptoms				
Pre-existing HT + Hypertensive crisis, *n* (%)	29 (15.9)	18 (13.4)	11 (22.9)	0.067^f^
New-Onset Hypertensive Attack, *n* (%)	20 (11)	16 (11.9)	4 (8.3)	
Pre-existing DM + Blood Glucose Regulation Disorder, n (%)	4 (2.2)	3 (2.2)	1 (2.1)	
New-Onset Blood Glucose Regulation Disorder, *n* (%)	1 (0.5)	1 (0.7)	0 (0)	
Non-Specific Pain, *n* (%)	106 (58.3)	75 (56.1)	31 (64.6)	
Size Increase in Incidental Mass, *n* (%)	22 (12.1)	21 (15.7)	1 (2.1)	
Clinical Diagnosis				
Incidentaloma, *n* (%)	48 (26.4)	33 (24.6)	15 (31.2)	0.067^f^
Pheochromocytoma, *n* (%)	45 (24.7)	27 (20.1)	18 (37.5)	
Cushing's Disease, *n* (%)	38 (20.9)	29 (21.6)	9 (18.7)	
Myelolipoma, *n* (%)	10 (5.5)	10 (7.5)	0 (0)	
Conn's Syndrome, *n* (%)	17 (9.3)	15 (11.2)	2 (4.2)	
Cancer, *n* (%)	15 (8.3)	13 (9.7)	2 (4.2)	
Cystic Lesion, *n* (%)	9 (4.9)	7 (5.3)	2 (4.2)	
History of Previous Surgery, *n* (%)	26 (14.3)	20 (14.9)	6 (12.5)	0.68^c^
Cholecystectomy	13	9	4	
Appendectomy	2	2	0	
Ascending Aorta Graft	1	1	0	
Cesarean Section	5	4	1	
Kidney Stone	2	1	1	
TAH + BSO	1	1	0	
Radical Hysterectomy	1	1	0	
Umbilical Hernia Repair	1	1	0	
ASA Score				
1, *n* (%)	14 (7.7)	13 (9.7)	1 (2.1)	** < 0.001**^**c**^
2, *n* (%)	96 (52.7)	80 (59.7)	16 (33.3)	
3, *n* (%)	72 (39.6)	41 (30.6)	31 (64.6)	
Performance Status, *n* (%)				
0, *n*(%)	136 (74.7)	112 (83.6)	24 (50)	** < 0.001**^**f**^
1, *n* (%)	32 (17.7)	22 (16.4)	10 (20.8)	
2, *n* (%)	13 (7.1)	0 (0)	13 (27.1)	
3, *n* (%)	1 (0.5)	0 (0)	1 (2.1)	
Additional Diseases				
Cardiac Disease, *n* (%)	28 (15.4)	14 (10.4)	14 (29.2)	**0.002**^**c**^
Pulmonary Disease, *n* (%)	16 (8.8)	12 (9)	4 (8.3)	0.896^f^
HT, *n* (%)	85 (46.7)	59 (44)	26 (54.2)	0.227^c^
DM, *n* (%)	46 (25.3)	28 (20.9)	18 (37.5)	**0.023**^**c**^
SVH, *n* (%)	4 (2.2)	3 (2.2)	1 (2.1)	0.716^f^
KBH, *n*(%)	3 (1.6)	3 (2.2)	0 (0)	0.567^c^
Imaging and Laboratory Findings				
Lesion Location				
Right, *n* (%)	91 (50)	70 (52.2)	21 (43.8)	0.313^c^
Left, *n* (%)	91 (50)	64 (47.8)	27 (56.3)	
Lesion Diameter (mm), (Median)(IQR)	42 (27.7–60)	40 (27–55.2)	46 (30.2–69.8)	0.34^m^
Presence of Functional Lesion, *n* (%)	95 (52.2)	68 (50.7)	27 (56.3)	0.512^c^

Bold values indicate statistically significant

*BMI* Body Mass Index, *IQR* Interquartile Range, *HT* Hypertension, *DM* Diabetes Mellitus, *TAH* + *BSO* Total Abdominal Hysterectomy + Bilateral Salpingo-Oophorectomy, *ASA* American Society of Anesthesiologists, *CVD* Cerebrovascular Disease, *CKD* Chronic Kidney Disease

^m^Mann–Whitney *U* Test

^c^Chi-square Test

^f^Fisher’s Exact Test

**Table 3:** Comparative analysis of intraoperative and postoperative data, as well as E-PASS scores between patients with and without postoperative complications after adrenalectomy

	Total (*n* = 182)	Presented with no postoperative complications (*n* = 134, % 73.6)	Presented with postoperative complications (*n* = 48, % 26.4)	*p*
Intraoperative Data				
Surgical Approach				
Laparoscopy, *n* (%)	101 (55.5)	92 (68.7)	9 (18.8)	** < 0.001**^**c**^
Open, *n* (%)	81 (44.5)	42 (31.3)	39 (81.3)	
Operation Time				
0–60 min, *n* (%)	56 (30.8)	52 (38.8)	4 (8.3)	** < 0.001**^**c**^
60–120 min, *n* (%)	90 (49.5)	71 (53)	19 (39.6)	
120–180 min, *n* (%)	23 (12.6)	7 (5.2)	16 (33.3)	
> 180 min *n* (%)	13 (7.1)	4 (3)	9 (18.8)	
Volume of Bleeding (ml) (Median)(IQR)	80 (60–122)	70 (50–90)	215 (120–300)	** < 0.001**^**m**^
Blood Transfusion Requirements, *n* (%)	12 (6.6)	0 (0)	12 (25)	** < 0.001**^**f**^
Reason for LA to OA Conversion, *n* (%)	37 (20.3)	16 (11.9)	21 (43.8)	** < 0.001**^**c**^
Bleeding	22	5	17	
Hemodynamic instability	5	4	1	
Insufficient field of view	10	7	3	
Postoperative Data				
Complication (Clavien–Dindo classification system)				
Grade 1, *n* (%)			26 (54.1)	
Grade 2, *n* (%)			12 (25)	
Grade 3a, *n* (%)			6 (12.5)	
Grade 3b, *n* (%)			2 (4.2)	
Grade 4a, *n* (%)			1 (2.1)	
Grade 4b, *n* (%)			1 (2.1)	
Grade 5, *n* (%)			0 (0)	
Hospital Stay (day) (Median)(IQR)	2 (2–3)	2 (2–3)	2 (2–3)	0.118^m^
Drain Use, *n* (%)	81 (44.5)	49 (36.6)	32 (66.3)	** < 0.001**^**c**^
E-PASS Score				
PRS (Median) (IQR)	0.3223 (0.2031–0.5328)	0.2672 (0.1963–0.4606)	0.5048 (0.2249–0.8023)	** < 0.001**^m^
SSS (Median) (IQR)	− 0.2278 (− 0.2587–0.1145)	− 0.2509 (− 0.2871–0.0725)	0.1462 (0.1044–0.181)	** < 0.001**^m^
CRS (Median) (IQR)	− 0.0677 (− 0.3077 to 0.1636)	− 0.1552 (− 0.3387 to [− 0.0087])	0.3059 (0.0115–0.4564)	** < 0.001**^m^

Bold values indicate statistically significant

*LA* Laparoscopic Adrenalectomy, *OA* Open Adrenalectomy, *E-PASS* Estimation of Physiologic Ability and Surgical Stress, *PRS* Preoperative Risk Score, *SSS* Surgical Stress Score, *CRS* Comprehensive Risk Score

^m^Mann–Whitney *U* Test

^c^Chi-square Test

^f^Fisher’s Exact Test

**Table 4:** Classification of postoperative complications according to the Clavien–Dindo classification system

Clavien–Dindo		*n* (%)
Grade 1	Complications that do not require pharmacological, surgical, endoscopic or radiological intervention	
	Wound infection requiring conservative management	9 (18.7)
	Severe pain	7 (14.6)
	Metabolic disorder	4 (8.4)
	Vomiting/Dyspepsia requiring medical intervention	3 (6.2)
	Bleeding/Hematoma requiring conservative treatment	3 (6.2)
Grade 2	Complications requiring pharmacological treatment	
	Bleeding requiring blood transfusion	12 (25)
Grade 3	Complications requiring surgical, endoscopic or radiological intervention	
Grade 3a	Complications that do not require general anesthesia	
	Wound infection requiring non-anesthetic for intervention	4 (8.3)
	Bleeding/Hematoma requiring non-anesthetic intervention	1 (2.1)
	Pain requiring non-anesthetic intervention	1 (2.1)
Grade 3b	Complications requiring general anesthesia	
	Ileus	1 (2.1)
	Hernia	1 (2.1)
Grade 4	Complications that are life-threatening and require intensive care	
Grade 4a	Single organ failure	
	Cerebrovascular event	1 (2.1)
Grade 4b	Multiple organ failure	
	Sepsis	1 (2.1)
Grade 5	Death due to complications	

In the group of patients who developed postoperative complications, the following parameters were significantly higher compared to the other group: BMI, ASA score, performance score, rates of heart disease and diabetes mellitus, open surgery rate, operation duration, amount of bleeding, need for blood transfusion, intraoperative complication rate, drain usage rate and E-PASS score parameters (PRS, SSS, and CRS). Demographic, clinical, radiological, laboratory, intraoperative, postoperative data and E-PASS scores for the patients are shown in Tables 2 and 3.

**Fig. 1:**
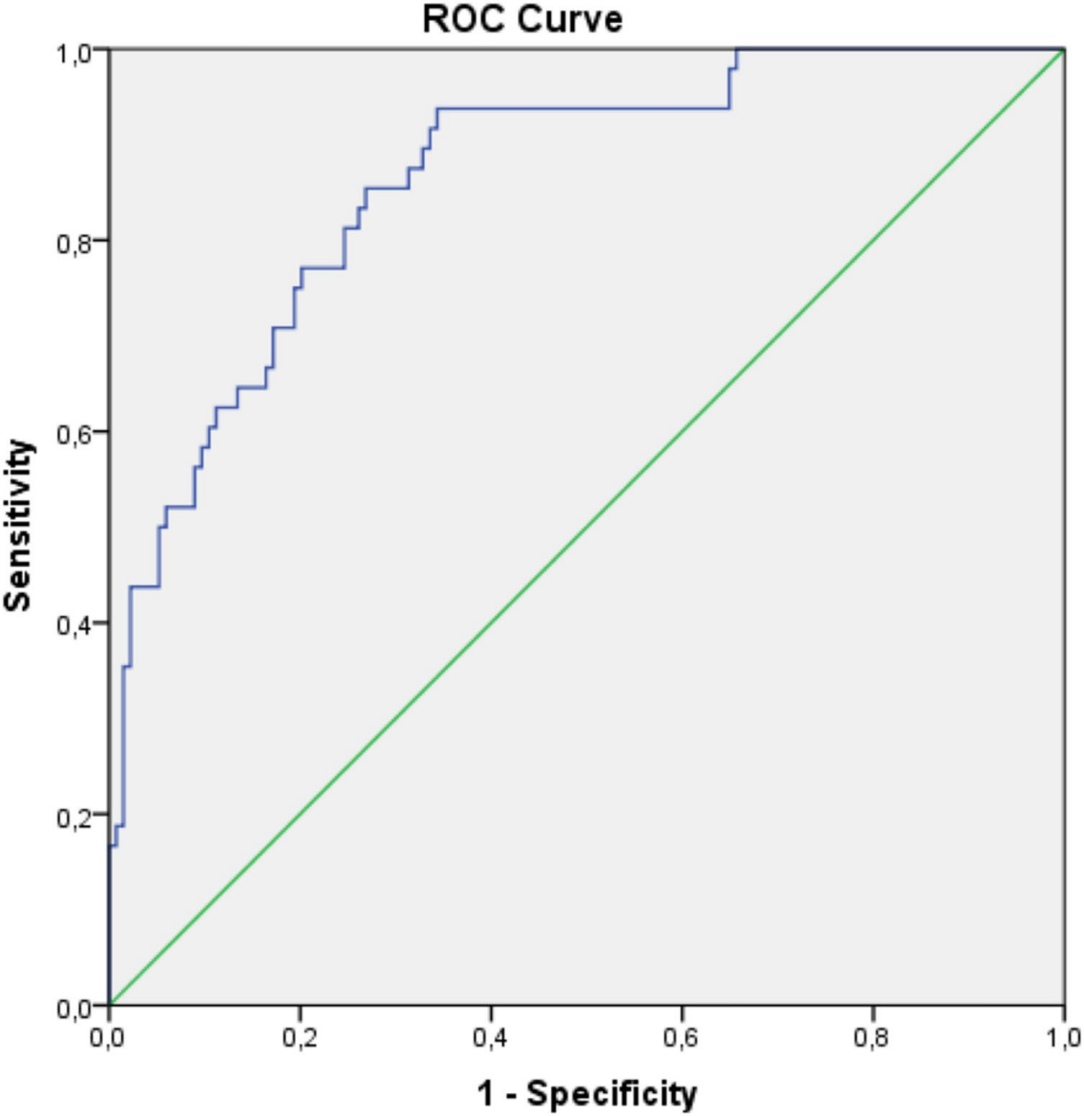
ROC analysis demonstrating the cut-off point for CRS evaluation

According to ROC curve analysis, the cut-off value for the CRS, which reflects the E-PASS score, in predicting postoperative complications was − 0.0677 (AUC = 0.866, CI 0.808–0.923; *p* < 0.001) (Fig. 1 and Table 5).

**Table 5:** The best cut-off point for CRS, which distinguishes the group with postoperative complications after adrenalectomy with a 95% confidence interval relative to the area under the ROC curve

	CRS
AUC	0.866
95% CI	0.808–0.923
p value	< 0.001
Cut-off point	− 0.0677
Sensitivity	0.938
Specificity	0.657

*CRS* comprehensive risk score, *AUC* area under curve, *CI* confidence interval

Postoperative complications in adrenalectomy were evaluated using multivariate logistic regression analysis. Accordingly, a higher BMI (OR = 1.264; 95% CI = 1.012–1.629; *p* = 0.021), longer operative time (OR = 4.199; 95% CI = 1.012–17.855; *p* = 0.049), increased bleeding (OR = 1.021; 95% CI = 1.012–1.03; *p* < 0.001) and an E-PASS CRS score > − 0.0677 (OR = 9.581; 95% CI = 2.356–38.957; *p* = 0.002) were identified as independent risk factors for postoperative complications (Table 6).

**Table 6:** Results of multivariate logistic regression analysis of postoperative complications and risk factors in patients undergoing adrenalectomy

Parameters	OR (95% CI)	*p*
Age (per year)	0.983 (0.934–1.035)	0.514
Female gender	1.824 (0.596–5.578)	0.292
BMI (per kg/m^2^)	1.394 (1.158–1.677)	** < 0.001**
History of previous surgery	1.266 (0.17–9.418)	0.818
ASA score > 1	2.865 (0.131–62.598)	0.503
Performance status > 1	1.464 (0.432–4.96)	0.541
Presence of cardiac disease	0.77 (0.14–4.285)	0.774
Presence of pulmonary disease	0.66 (0.067–6.518)	0.723
Presence of HT	0.66 (0.175–2.482)	0.538
Presence of DM	1.589 (0.368–6.873)	0.535
Presence of CVD	3.821 (0.141–103.333)	0.426
Lesion location (ref. right)	1.232 (0.372–4.082)	0.733
Lesion diameter (per mm)	0.979 (0.956–1.003)	0.083
Presence of functional lesion	0.635 (0.177–2.273)	0.485
Surgical approach (ref. open)	1.445 (0.168–12.423)	0.737
Operation time (per min)	4.199 (1.012–17.855)	**0.049**
Volume of bleeding (per mL)	1.021 (1.012–1.03)	** < 0.001**
E-PASS CRS > −0.0677	6.17 (2.365–9.991)	** < 0.001**

Bold values indicate statistically significant

*CI* Confidence Interval, *BMI* Body Mass Index, *E-PASS* Estimation of Physiologic Ability and Surgical Stress, *CRS* Comprehensive Risk Score, *ASA* American Society of Anesthesiologists, *HT* Hypertension, *DM* Diabetes Mellitus, *CVD* Cerebrovascular Disease, *ref* reference

## Discussion

The incidence of perioperative complications in adrenalectomy is reported to range from 1.7 to 30.7% [16, 17]. To predict the risk of complications after adrenalectomy, the PACS (Arterial pressure, surgical approach, catecholamine level, sex) risk score and the National Surgical Quality Improvement Program (NSQIP) risk calculator have been successfully utilized. In addition, various nomograms have been developed [8–10]. Although various risk calculators and nomograms are used to predict adrenalectomy complications, these studies have primarily focused on specific surgical types or adrenal lesions [9, 10]. Additionally, while various predictive models for adrenalectomy exist in the literature, it has been observed that they do not thoroughly examine preoperative and intraoperative factors. The E-PASS scoring system comprehensively evaluates multiple preoperative and intraoperative factors, enabling the early prediction of complications in the immediate postoperative period [14].

The E-PASS scoring system has been successfully applied in surgeries across various surgical specialties [12–14, 18, 19]. Kondo et al. reported that an E-PASS CRS > − 0.058 is associated with an elevated risk of postoperative complications in colorectal surgeries [18]. Additionally, it has been found that the E-PASS scoring system can successfully predict complications in spinal surgery and acetabular fracture surgery [12, 19]. In the literature, there are two studies that investigate the use of E-PASS in urological surgery [13, 14]. In the study involving 252 patients undergoing radical cystectomy and ileal loop surgery, the cut-off value for the CRS predicting postoperative complications was established at 0.4911 [14]. Additionally, a study has been conducted on laparoscopic nephrectomy. According to the findings of this research, the CRS > − 0.2996 is associated with a 2.8 times increase in the risk of postoperative complications [13]. This study is the first in the literature to examine adrenalectomy and it found that an E-PASS CRS score > − 0.0677 was identified as an independent risk factor for postoperative complication.

Surogi et al. examined the complication rates in 154 cases of OA and LA. The total complication rate was 26%, with complications distributed as 67.5% in Grade I and Grade II, 15% in Grade IIIa, 7.5% in Grade IIIb, 0% in Grade IV and 10% in Grade V according to the CDC classification [7]. Parente et al. in their multicenter study, analyzed 406 cases of OA and LA and reported a complication rate of 36.4%. Of these complications, 60.8% were Grade I and Grade II, 0.7% were Grade IIIa, 2.1% were Grade IIIb, 35.1% were Grade IVa and 1.3% were Grade V [9]. In our study, the total complication rate was 26.4%, with 18.8% of the complications classified as major (Grade IIIa and above). We believe that the lower overall and major complication rates in our study compared to the multicentric study are due to the fact that the data in our study were analyzed from a single surgeon's cases.

There are varying findings in the literature regarding the effect of BMI on the development of postoperative complications. Hauch et al. in their study examining 7829 adrenalectomy cases, identified obesity (BMI ≥ 30 kg/m^2^) as a significant risk factor for postoperative complications [20]. In another study examining 9820 adrenalectomy cases, a BMI of ≥ 40 kg/m^2^ was reported to represent a significant risk [4]. In cases where laparoscopic surgery was performed, BMI was not found to present a risk for transperitoneal procedures, but it was identified as a risk factor for retroperitoneal surgery [5, 6, 10]. In our study, due to the investigation of the E-PASS scoring system, laparoscopic (transperitoneal) and OA cases were examined as a whole, with BMI identified as an independent risk factor for postoperative complications (OR = 1.394; 95% CI = 1.158–1.677; *p* < 0.001).

Adhesions resulting from previous abdominal surgery can cause prolonged operative times, increased risks during the initial entry into the abdominal cavity in laparoscopic surgery, and an increase in intraoperative complications [21]. However, Morris et al. in their retrospective study of 246 LA cases found no significant difference in perioperative complication rates when comparing patients with a history of prior upper abdominal surgery to those without such a history [22]. Additionally, Mazeh et al. found that adhesions were more prevalent in cases with a history of ipsilateral and contralateral upper abdominal as well as lower abdominal surgeries. However, they observed no differences in intraoperative bleeding or the rate of conversion from LA to OA [23]. Our study also found that a history of prior abdominal surgery was not a predictor of postoperative complications. However, it should be noted that some of the LA cases we examined did involve conversion to OA.

Studies have produced varying results regarding the side of adrenalectomy and the risk of complications. In a multivariable logistic regression analysis of 462 patients, left-sided adrenalectomy was identified as an independent risk factor for postoperative complications [24]. There are also studies reporting that the side of the operation is not a risk factor for complications [25, 26]. However, the general consensus is that greater attention should be given to parenchymal and vascular structures in left adrenalectomy [3, 24]. In our study, no significant association was found between the surgical side and the risk of complications.

Adrenal surgery has been associated with a high ASA score as a significant factor in the development of postoperative complications [5, 6, 27]. Chen et al., in their study of 653 LA cases, found an increase in perioperative complications and length of hospital stay in patients with an ASA score of 3 or higher [5]. Gupta et al. examined 988 LA cases and identified that, in addition to the development of postoperative complications, the operation time was longer in the ASA 4 group [27]. The influence of performance status on surgical outcomes is well-established, with evidence showing that patients with restricted abilities in daily living activities tend to experience longer hospital stays and higher rates of morbidity and mortality [28]. However, research specifically on adrenalectomy surgery in this topic is limited [27]. In our study, it was found that, similar to existing information, a high ASA score and ECOG performance score present a risk for the development of postoperative complications (*p* < 0.001). Additionally, no difference in hospital stay was observed in the group with complications in our study, which is attributed to the low rate of major complications.

The Charlson Comorbidity Index is a scoring system that takes into account both the patient's age and the presence of additional chronic diseases [29]. It provides a comprehensive assessment of comorbidities, which is particularly useful in evaluating surgical risks [29]. Numerous studies have examined adrenalectomy complications using this index, highlighting its importance in predicting postoperative outcomes in patients undergoing adrenal surgery [4, 7, 30]. Although age and chronic diseases were examined in detail in our study, the Charlson Comorbidity Index was not used. Cardiac disease and diabetes were identified as significant risk factors for the development of postoperative complications (*p* = 002, *p* = 0.023 respectively). The fact that age was not found to be a risk factor for postoperative complications may be attributed to the limited number of patients > 65 years in our study.

In the literature, it has been reported that the final histopathology result of adrenalectomy generally does not affect postoperative complications [6, 10, 31]. Mellon et al. did not find statistically significant differences in the development of postoperative complications among surgeries for PHEO, CS, CuS, malignant adrenal disease and nonfunctioning adenomas [31]. Hattori et al. in their study of 265 unilateral LA cases found that the final pathology of CS, PHEO and CuS did not represent a risk for the development of postoperative complications [6]. In another study involving 610 patients, no differences were observed in the development of postoperative complications among cases with histopathology of adenoma, hyperplasia and PHEO [10]. In our study, it was observed that consistent with the literature neither the nature of the mass nor the presenting symptom represented a risk for postoperative complications. We believe that this result is due to the stabilization of patients in the preoperative period through premedication in cases of functional masses.

Large adrenal masses (> 6 cm) have been determined to be associated with increased surgical duration and a higher risk of intraoperative events during LA [5, 32]. Additionally, various studies have reported that a lesion diameter greater than 5–6 cm is an independent predictor for conversion from LA to OA [33, 34]. In our study, since OA was preferred for lesions larger than 6 cm, lesion diameter was not a significant factor in the development of postoperative complications. However, in our study, complication rates in the OA group were found to be statistically significantly higher than those in the LA group (*p* < 0.001). This may be due to the preference for OA in complicated cases, as well as the conversion to OA during LA in the event of intraoperative bleeding, inadequate field of view or hemodynamic instability. Additionally, the higher complication rate in patients with drain usage was thought to be related to the more frequent use of drains in OA procedures (*p* < 0.001).

## Limitations

The retrospective design of the study is a limitation, and the single-center nature of the study may limit the generalizability of the findings to other institutions with different practices. Furthermore, the exclusion of other risk assessment tools, such as the Charlson Comorbidity Index, could affect the comprehensiveness of the risk evaluation. However, the use of the Charlson Comorbidity Index is not required according to the E-PASS scoring system.

The higher complication rate observed in the open adrenalectomy group may have been influenced by the preference for this approach in more complex cases. This preference potentially affects the results. Nevertheless, it should be noted that the E-PASS scoring system allows for the evaluation of both laparoscopic and open cases together [11].

## Conclusion

The E-PASS scoring system, a model that can be easily applied using preoperative and intraoperative data, is able to predict postoperative complications for adrenalectomy. Its application in clinical practice can assist surgeons in identifying high-risk patients, allowing for improved perioperative management and potentially reducing the incidence of adverse outcomes.

## Data Availability

The present data belong to and are stored at the Baskent University Adana Dr. Turgut Noyan Application and Research Center and cannot be shared without permission.
